# Ecophysiological and Biochemical Responses Depicting Seed Tolerance to Osmotic Stresses in Annual and Perennial Species of *Halopeplis* in a Frame of Global Warming

**DOI:** 10.3390/life12122020

**Published:** 2022-12-03

**Authors:** Aysha Rasheed, Sarwat Ghulam Rasool, Pilar Soriano, Elena Estrelles, Bilquees Gul, Abdul Hameed

**Affiliations:** 1 Dr. Muhammad Ajmal Khan Institute of Sustainable Halophyte Utilization, University of Karachi, Karachi 75270, Pakistan; 2 Cavanilles Institute of Biodiversity and Evolutionary Biology, Botanic Garden, University of Valencia, Quart, 80, 46008 Valencia, Spain

**Keywords:** germination ecophysiology, biochemical responses, seed stress tolerance, climate change, *Halopeplis*

## Abstract

Plant abundance and distribution are regulated by subtle changes in ecological factors, which are becoming more frequent under global climate change. Species with a higher tolerance to such changes, especially during early lifecycle stages, are highly likely to endure climate change. This study compared the germination adaptability of *Halopeplis amplexicaulis* and *H. perfoliata*, which differ in life-form and grow in different environments. Optimal conditions, tolerances and the biochemical responses of seeds to osmotic stresses were examined. Seeds of *H*. *perfoliata* germinated in a wider range of temperature regimes and were more tolerant to osmotic stresses than *H. amplexicaulis* seeds. Neither NaCl nor PEG treatment invoked the H_2_O_2_ content in germinating seeds of the tested species. Consequently, unaltered, or even decreased activities of H_2_O_2_ detoxification enzymes and non-enzymatic antioxidants were observed in germinating seeds in response to the aforementioned stresses. High and comparable levels of recovery from isotonic treatments, alongside a lack of substantial oxidative damage indicated that the osmotic stress, rather than the ionic toxicity, may be responsible for the germination inhibition. Hence, rainy periods, linked to water availability, may act as a key determinant for germination and *H. perfoliata* could be less affected by global warming owing to better germinability under high temperatures compared with *H. amplexicaulis*. Such studies involving biochemical analysis coupled with the germination ecology of congeneric species, which differ in life-form and occurrence are scarce, therefore are important in understanding the impacts of global changes on species abundance/distribution.

## 1. Introduction

Salt-affected habitats are complex environments in which subtle changes in ecological factors determine plant occurrence, communities’ richness and distribution [1]. In this sense, differences in levels of germination success linked to salt tolerance and biotic interactions during seedling emergence and establishment are important in plant composition and salt marsh zonation patterns [2]. Therefore, the knowledge of biological traits related to reproductive behaviour is essential for the development of conservation strategies and the management of threatened species [3] and ecosystems, such as salt marshes.

Water/osmotic stress caused by a decrease in osmotic potential under salinity and drought inhibits various plant processes, especially seed germination that determines plants dispersal and abundance [1,4]. In addition to osmotic effect, salinity stress leads to an accumulation of ions, which can produce ionic stress [4]. In both cases, salinity limits plant growth and development required for survival by reducing turgor pressure and photosynthesis [5]. These water/osmotic stresses activate diverse physiological and biochemical response mechanisms in plants linked to seed traits, morphology and anatomy, water relation, or antioxidative metabolism [6,7].

Evidences suggest that the exposure of seeds to osmotic stresses such as salinity and drought can inflict enhanced production of potentially toxic reactive oxygen species (ROS), which if not quenched efficiently through cellular antioxidants can induce oxidative damage to important cellular components, such as membrane lipids, proteins and nucleic acids [8,9,10]. This is particularly true for the seeds of halophytes which experience large variations in soil moisture and salinity; therefore, the success of their seed germination and seedling establishment depends on minimizing the damages resulting from enhanced ROS under the aforementioned stresses [11,12]. The antioxidant machinery of the plant cells is composed of many enzymatic and non-enzymatic components [11,12]. Superoxide dismutase (SOD), catalase (CAT) and ascorbate peroxidase (APX) are key antioxidant enzymes, whereas ascorbic acid (AA) and glutathione (GSH) are common non-enzymatic antioxidants [8,13,14]. Most information on ROS homeostasis in plants under environmental stresses is based on vegetative tissues and any knowledge about ROS production and quenching in germinating seeds, particularly of halophytes, is generally scant [8,11,12].

This study examined germination behaviour, seed micromorphology and biochemical responses of two species of the genus *Halopeplis*; viz., *H. amplexicaulis* and *H. perfoliata*. *Halopeplis* is a characteristic genus of the leaf succulent eu-halophyte Amaranthaceae [15], which includes only three species, *H. perfoliata* (Forssk.) Bunge, *H. pygmaea* (Pall.) Bunge and *H. amplexicaulis* (Vahl) Ung.-Sternb. *Halopeplis amplexicaulis* is an annual found in salty habitats, throughout the Mediterranean basin and other territories in Africa, including southern Africa where the populations were presumably introduced years ago [16], but are currently cited as a native plant [17]. It is the only European and Mediterranean representative species of this genus. Whereas *H. perfoliata* is a chamephyte species which inhabits coastal salt marshes of the Arabian Peninsula [18]. It is an example of a species with a typical circum-Arabian distribution in the Nubo-Sindian zone of the Sahara-Sindian phytochorion [19].

Some earlier studies have reported different ecological aspects of the seed germination of the *Halopeplis* species individually [20,21,22,23,24]. However, a comparison of the osmotic stresses and biochemical aspects of the seed germination of the *Halopeplis* species has not been done. In this context, this study deals with the comparative seed germination strategies and biochemical responses to osmotic stresses (NaCl and PEG) of two species of *Halopeplis* with different life-forms and which grow in different environmental conditions. Furthermore, studies involving biochemical analysis coupled with the germination ecology of congeneric species differing in life-form and occurrence are limited but crucial to understand species abundance/distribution in a changing climate. In this sense, our study attempted to approach these aspects, which have so far not been sufficiently clarified, through the following objectives:

(1) To study whether the different environmental conditions of the sample sites, especially a more stressful maternal environment, direct the plasticity of seed responses to temperature, salt and drought tolerance.
(2) To test whether ionic toxicity is a relevant aspect that affects germination in saline conditions.
(3) To confirm the relation of life-form and germination responses.
(4) To evaluate ROS production and quenching responses of the germinating seeds of the two species under osmotic stresses (NaCl and PEG).
(5) To unveil similarities in ROS production and quenching responses of germinating seeds of the two species.
(6) To assess if species from a more stressful environment have a greater ability to survive in a global warming scenario.

Additionally, seed features were characterized in order to determine a potential relationship between germination behaviours under different osmotic stresses.

## 2. Materials and Methods

**Figure 1:**
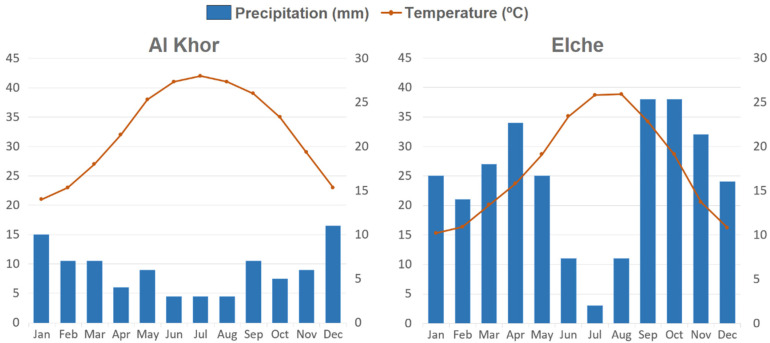
Climate diagrams for the two populations.

- Seeds of *H. perfoliata* and *H. amplexicaulis* were collected from natural populations at the time of dispersal. After collection, seeds were cleaned and stored in paper bags in a controlled environment (20 °C and 40–50% RH) until used in the germination tests.
- Seeds of *H. amplexicaulis* were gathered from El Hondo Natural Park (38°09′57.4″ N 0°42′39.1″ W), an inland-protected lagoon situated on the east coast of Spain in Mediterranean xeric bioclimate (T. 18 °C; P: 286 mm) (Figure 1). The EC values measured during the driest season were between 67.3 and 81.5 mmhos cm^−1^ which corresponded to a salt concentration range of 0.693 M to 0.892 M.
- Seeds of *H. perfoliata* were collected from a salt marsh near the coast of Al khor, Qatar (25°45′18.1″ N 51°31′44.7″ E) in tropical desertic bioclimate (T: 33 °C; P: 88 mm) (Figure 1). Soil EC values of the study area ranged between 31.5 to 187.7 mmhos cm^−1^ [25].

### 2.1. Seed Morphological Features

Seed shape was described by following the scheme of [26]. Dimensions, width and length were measured using the image analysis software ImageJ (National Institutes of Health, Bethesda, MD, USA) [27]. The weight of 100 seeds was determined using an Orion Cahn C-33 microbalance. The scanning electron microscopy (SEM) aspects of the seed coat were also examined. Seed surface was analysed with a SEM Hitachi S-4100 (Hitachi Scientific Instruments, Mountain View, CA, USA), at the Central Service for Experimental Research (SCSIE), Electronic Microscopy Section of the University of Valencia. Samples were mounted on aluminium stubs with a carbon double-sided tape, were sputter coated with a 100–200 Å thick layer of gold and palladium by an SC500, Bio-Rad sputter coater (Bio-Rad, BioRad Laboratories Pty Ltd., New South Wales, Australia), and were examined at an accelerating voltage of 5 kV. Seed micrographs at different magnifications were used to determine the seed coats’ morphological features. The patterns observed were given descriptive terms according to their appearance at different magnifications [28].

### 2.2. Germination Tests

Germination tests were carried out in plastic Petri dishes (50 mm diameter × 9 mm depth), which were kept in temperature-controlled cabinets. The final germination percentage was calculated after 20 days.

Seed tolerance was tested at seven increasing NaCl concentrations, 0, 100, 200, 300, 400, 500 and 600 mM and six isotonic solutions of polyethylene glycol 6000 (PEG), at four different alternating temperature regimes (20/10, 25/15, 30/20 and 35/25 °C), using daylight fluorescent illumination with a 12 h photoperiod (100 µmol·m^−2^·s^−1^). There were four replications of 25 seeds each per treatment.

The tolerance index (TI) was calculated for each treatment as the observations under stress divided by the means of the controls [29].

Seeds which did not germinate under osmotic treatments were transferred to distilled water and maintained for 15 days to check the recovery capacity. Recovery germination percentage was determined using the equations given in [30].

The first (FD) and the last (LD) germination days and the germination periods (GP), as the number of days between first and final germination, were also calculated for all treatments, including recovery tests. Germination rate (GR) was calculated using a modified Timson’s index [31]: ∑G/t, where G is the percentage of seed germination at 2-day intervals and t is the number of measurement periods.

### 2.3. Biochemistry Tests

#### 2.3.1. Seed Water Uptake

Relative water uptake was determined by following the method of [32] with slight modifications. Briefly, seeds (50 mg) were immersed in distilled water (control), −0.88 MPa solution of NaCl (salt) and iso-osmotic solution PEG-6000 (drought). An increase in weight of seeds was recorded as soon as radicle emergence commenced in control (i.e., 20 h for *H. perfoliata* and 40 h for *H. amplexicaulis*). The relative increase in seed fresh weight (Wr) due to imbibitional water uptake was calculated using the following formula: Wr (%) = (Wf − Wi)/Wi × 100, where Wi is the initial weight of seeds and Wf is the weight after imbibition in test solution [32].

#### 2.3.2. Mitochondrial Activity 

The mitochondrial respiratory activity of the germinating seeds of test species was estimated by following the slightly modified method of [33]. Seeds of *H. perfoliata* and *H. amplexicaulis* were soaked in the aforementioned test solutions for 20 and 40 h, respectively. Afterwards, seeds were cut into two halves with the help of a sharp razor blade and incubated in 1% (*w*/*v*) 2,3,5-triphenyl tetrazolium chloride (TTC) solution prepared in 50 mM phosphate buffer (pH 7.3) under dark conditions for 24 h at room temperature. Red-colored triphenyl formazan, which is the indicator of mitochondrial respiratory activity, was extracted in 100% ethanol and estimated by recording absorbance at 520 nm. Mitochondrial activity was expressed as ΔA500 mg^−1^ FW.

#### 2.3.3. Hydrogen Peroxide and Malondialdehyde 

Hydrogen peroxide (H_2_O_2_) content of germinating seeds was measured by using the KI reagent method of [34]. Whereas the extent of lipid peroxidation was measured by quantifying the malondialdehyde (MDA) content according to the method of [35].

#### 2.3.4. Enzymatic Antioxidants

Antioxidant enzymes of the seeds were extracted by following the method described in [8]. The activity of superoxide dismutase (SOD; EC 1.15.1.1) was assayed according to the method of [36]. Catalase (CAT; EC 1.11.1.6) activity was measured by following the method of Aebi (1984). The activity of guaiacol peroxidase (GPX, EC 1.11.1.7) was assessed according to [37]. Ascorbate peroxidase (APX, EC 1.11.1.11) activity was estimated by following [38]. Glutathione reductase (GR, EC 1.6.4.2) activity was measured through the method of [39]. Protein content of the extracts was estimated according to the [40] method. Enzyme activities were expressed as units of enzyme activity per milligram protein.

#### 2.3.5. Non-Enzymatic Antioxidants 

The ascorbate (AsA) and dehydroascorbate (DHA) contents of the seeds were quantified according to the method of [41]. Whereas, reduced (GSH) and oxidized (GSSG) forms of glutathione were estimated by following the method of [42].

### 2.4. Statistical Analysis

All data were expressed as mean values ± SD (standard deviation) and were statistically analysed using the SPSS 16.0 software (SPSS, Chicago, IL, USA). A Student’s *t*-test for independent samples was used to compare seed features (95% confidence interval). Germination percentages were arcsine-transformed before the analysis to approximate the normality assumption for analysis of variance [43]. A three-way ANOVA was applied to determine the effects of species, stress treatments and temperature regimes on germination percentages and velocity, in both stress and recovery tests. Graphical visualization boxplots were depicted showing that the probability of values are included within a specific range, and the median as the box separators (*p* < 0.005).

## 3. Results

### 3.1. Seed Morphological Features

Seeds were broadly ovate in *H. amplexicaulis* to narrowly ovate in *H. perfoliata*, with a section in both species from compressed (1:2) to flattened (1:3). When seed traits were analysed, significant differences were found in all the parameters measured. Seeds of *H. perfoliata* are longer than those of *H. amplexicaulis* and values of length/weight ratio are also higher in the case of *H. perfoliata*. Conversely, seeds of *H. amplexicaulis* are wider and heavier than *H. perfoliata* seeds (Table 1).

**Figure 2:**
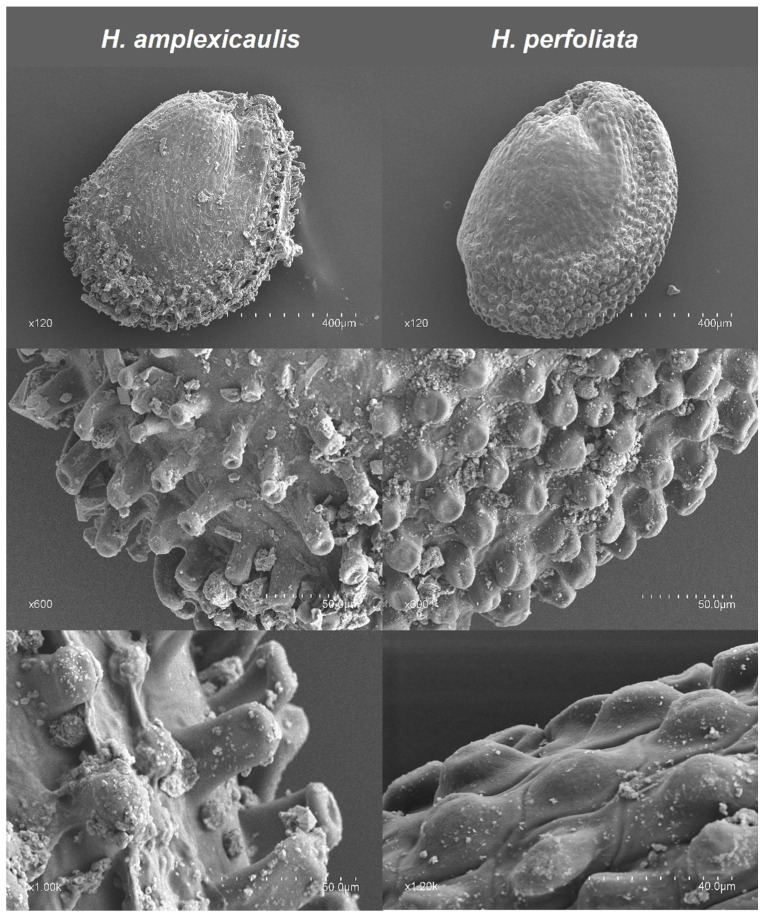
Ultra-sculpture of seed surface using scanning electron microscopy (SEM).

Regarding seed coat morphology under SEM examination, the surface was smooth, except for the external side of the seed, where the outer epidermal periclinal walls of the cells were convex, showing a differential sculpture over the radicular ridge. The outline of cells was irregularly elongated, sometimes prismatic. Both species had papillae aligned along the dorsal side in a more or less wide area over the seed’s outer edge, above the radicle of the embryo (Figure 2). However, differences between species were appreciable in the size of the papillae. Seeds of *H. perfoliata* were covered with papillae, more or less softly rounded, smaller, wider than long (14–15 × 22–25 µm) and sometimes these papillae were flattened. In contrast, *H. amplexicaulis* seeds had more elongated cylindrical projections, were higher and thinner (35–39 × 13–17 µm) and better adjusted to a tuberculated pattern.

### 3.2. Germination Tests

**Figure 3:**
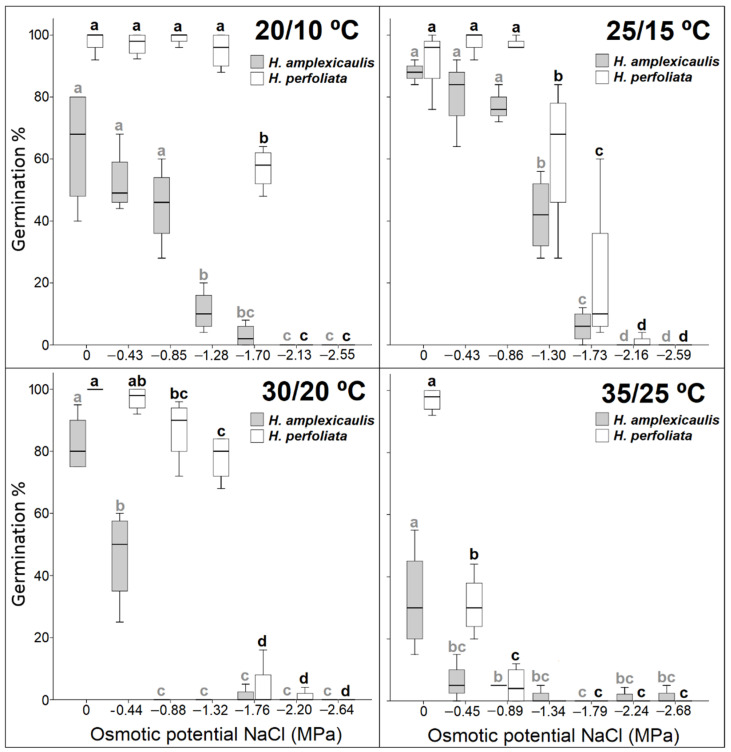
Seed germination percentage of tested species under increasing NaCl concentrations at the different temperature regimes. Bars with different alphabets are significantly different from each other (LSD; *p* < 0.05).

Seeds of *H. perfoliata* showed a wider positive response in terms of temperature and concentration range than *H. amplexicaulis* seeds. Overall, *H. perfoliata* seeds showed a higher germination percentage over a wide range of temperatures, including the lowest temperature regimes. Whereas the less tolerant species, *H. amplexicaulis*, reached its optimum at a 25/15 °C (Figure 3).

Seeds of *H. amplexicaulis* germinated in up to −1.30 MPa under the optimal temperature (25/15 °C), but germination was not higher than 42%. However, *H. perfoliata* seeds could germinate even in −1.70 MPa, with values higher than 55% at 20/10 °C (Figure 3; Table 2).

Germination periods (number of days between first and final germination) were shorter for *H. perfoliata* in all treatments, which ranged between 2 and 5 days in this species compared to the range of 1 and 16 days in *H. amplexicaulis*. A noteworthy increase in the number of days to the first germination was observed, especially in *H. amplexicaulis*, with increases in osmotic strength at all temperatures considered (Table 2).

**Figure 4:**
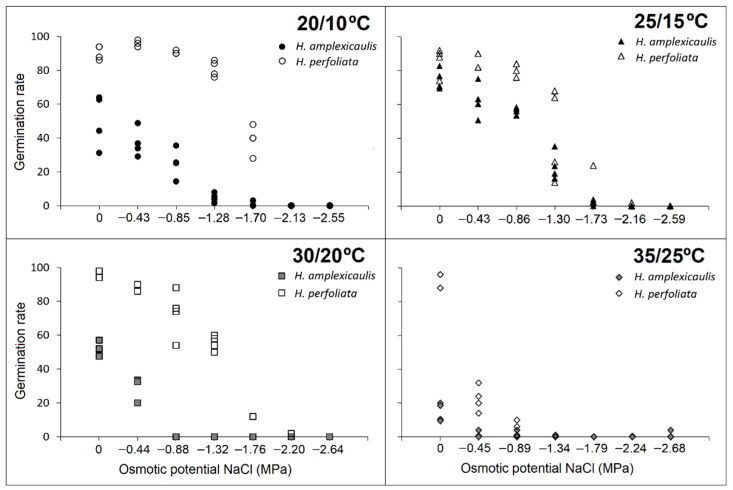
Regression plots for the germination rate (Timson index, maximum 100) of seeds under increasing NaCl concentration at different temperature regimes.

Germination rate showed that, as a rule, the rate of germination decreased, in both species, with an increase in salt concentration. This decrease was more pronounced in *H. amplexicaulis* compared with *H. perfoliata*. The observed inhibitory effect of salt concentration on the germination rate was greater at the highest temperature regime, 35/25 °C, in both species (Figure 4).

**Figure 5:**
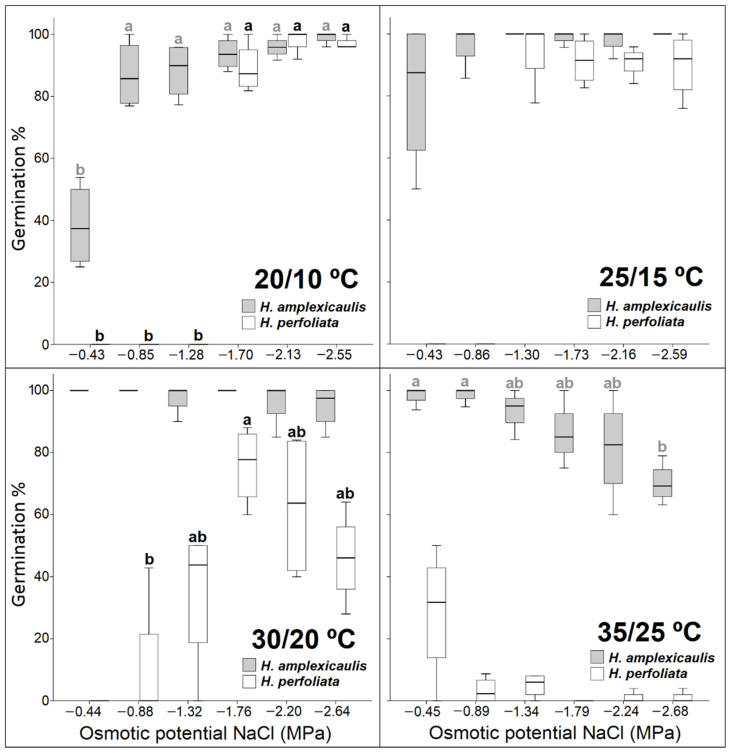
Recovery test of germination after transfer from NaCl to water in different temperature regimes. Bars with different alphabets are significantly different from each other (LSD; *p* < 0.05).

After the salinity treatments, when un-germinated seeds were transferred to distilled water, germination percentages increased with an increase the in pre-transfer salinity concentration. This behaviour was especially evident in *H. amplexicaulis*, where an increase in germination compared to the control was observed, practically, at all the temperatures tested (Figure 3 and Figure 5).

Nevertheless, when observing the recovery percentages of *H. perfoliata*, higher germination recovery values in salt compared with those obtained in the control were not achieved. Only at the lower temperatures the germination recovery values were equal to the control (Table 3 and Table 4; Figure 3 and Figure 5).

Regarding the germination period in the recovery tests, a remarkable decrease in the time until the beginning of germination was noted in *H. amplexicaulis*. In most cases, the first day of germination corresponded to the day after sowing, even at the higher temperatures and at the higher salt concentrations. Moreover, a decrease in the germination period during recovery was observed in *H. amplexicaulis* compared with the salt treatments (Table 2 and Table 3).

The rate of germination recovery indicated a different behaviour in the two compared species. *Halopeplis amplexicaulis* reached high values, at all the concentrations and temperatures tested, exceeding, in almost all cases, the values of the control. Whereas *H. perfoliata* seeds obtained high values only in the highest salt concentrations and at the lowest temperatures tested (Table 4 and Table 5).

**Figure 6:**
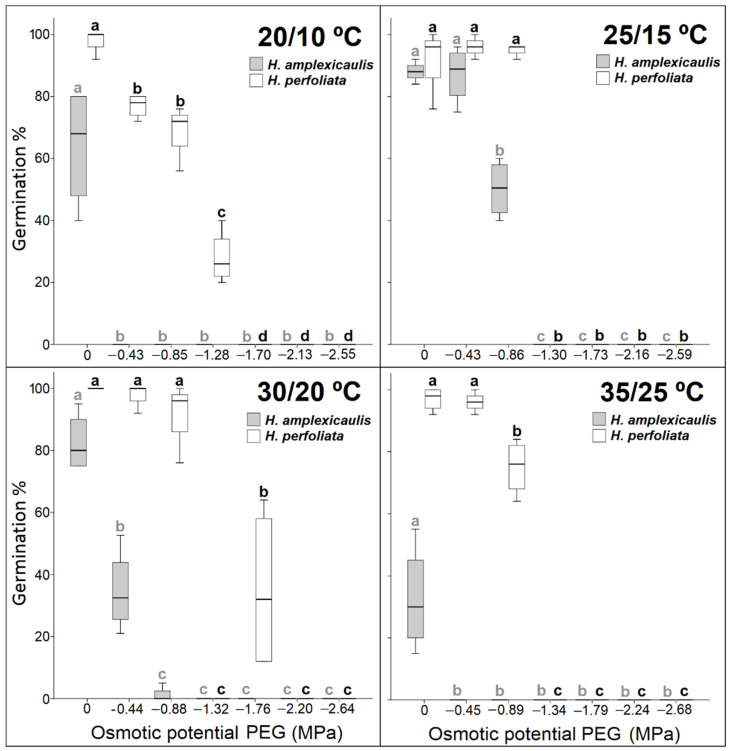
Germination percentages under different concentrations of polyethylene glycol and different temperature regimes. Bars with different alphabets are significantly different from each other (LSD; *p* < 0.05).

Concerning germination results of both species in isotonic solutions of PEG, the same behaviour pattern was observed as in the case of the NaCl treatments (Figure 6). In all the concentrations and temperatures, germination percentages were higher in *H. perfoliata* than in *H. amplexicaulis*.

Higher germination percentages were achieved in both species with NaCl than in PEG, except for the highest tested temperatures in *H. perfoliata*, where the germination percentage in PEG was higher than in NaCl.

**Figure 7:**
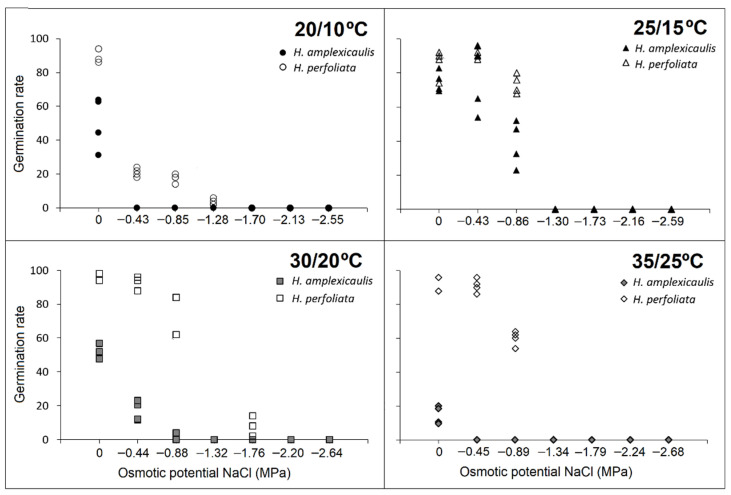
Regression plots for the rate of seed germination (Timson index, maximum 100) for both studied species under increasing PEG concentration and different temperature regimes.

The germination rate revealed that, as in the case of salt treatments, the rate of germination generally decreased, in both species, with an increase in PEG concentration. The observed inhibitory effect on the germination rate was greater in *H*. *amplexicaulis*, especially at the highest temperature regime (Figure 7). The preference for the temperature 25/15 °C that was already observed in the salt tests was confirmed. 

**Figure 8:**
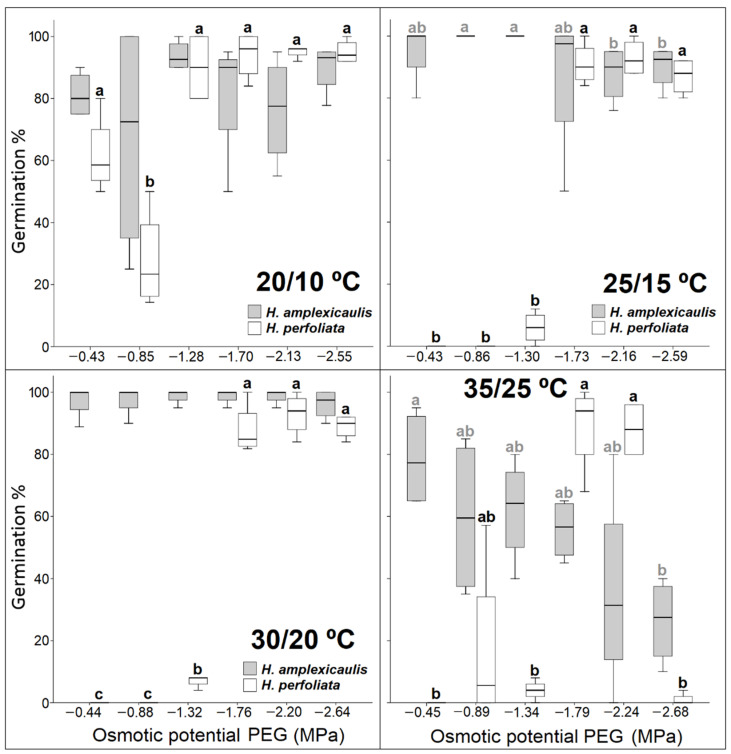
Recovery data after the treatment with PEG in different temperature regimes. Bars with different alphabets are significantly different from each other (LSD; *p* < 0.05).

Recovery data after the treatment with PEG were similar to those obtained in the recovery tests after exposing seeds to increasing NaCl concentrations. In *H. amplexicaulis*, germination percentages were equal to or above the control in almost all the tests. However, although the results were better in *H. perfoliata* at the higher temperatures, in comparison with the treatments with salt, in no case did these values surpass the value of the control (Figure 8).

The rate for recovery data followed a more or less similar pattern to that observed in the case of salt recovery. Except for the temperature of 35/25 °C, *H. amplexicaulis* reached high values at all concentrations whereas *H. perfoliata* obtained better percentages at high PEG concentrations under higher temperatures than in the treatment with NaCl (Table 6 and Table 7).

The best results in terms of tolerance were obtained in *H. perfoliata.* The values of the tolerance index (TI) exceed 80%, at osmotic potentials from −0.85 to −0.89 MPa, depending on temperature regime and 60% at values from −1.28 to −1.34 MPa, almost in all the temperatures tested (except 35/25 °C). Similar results were obtained in the treatment with PEG, where TI values for *H. perfoliata,* at values from −0.85 to −0.89 MPa, did not drop below 70% at any of the temperature regimes. On the other hand, the *H. amplexicaulis* only reached TI values higher than 70%, at the same osmotic potentials, in the case of salt treatments and at optimum temperature (25/15 °C), but in none of the PEG concentrations tested (Table 8).

The three-way ANOVA comparing the effects of all the treatments showed that germination percentage and rate were significantly affected by species, temperature, salt concentration and their interactions (Table 9).

### 3.3. Biochemistry Tests

**Figure 9:**
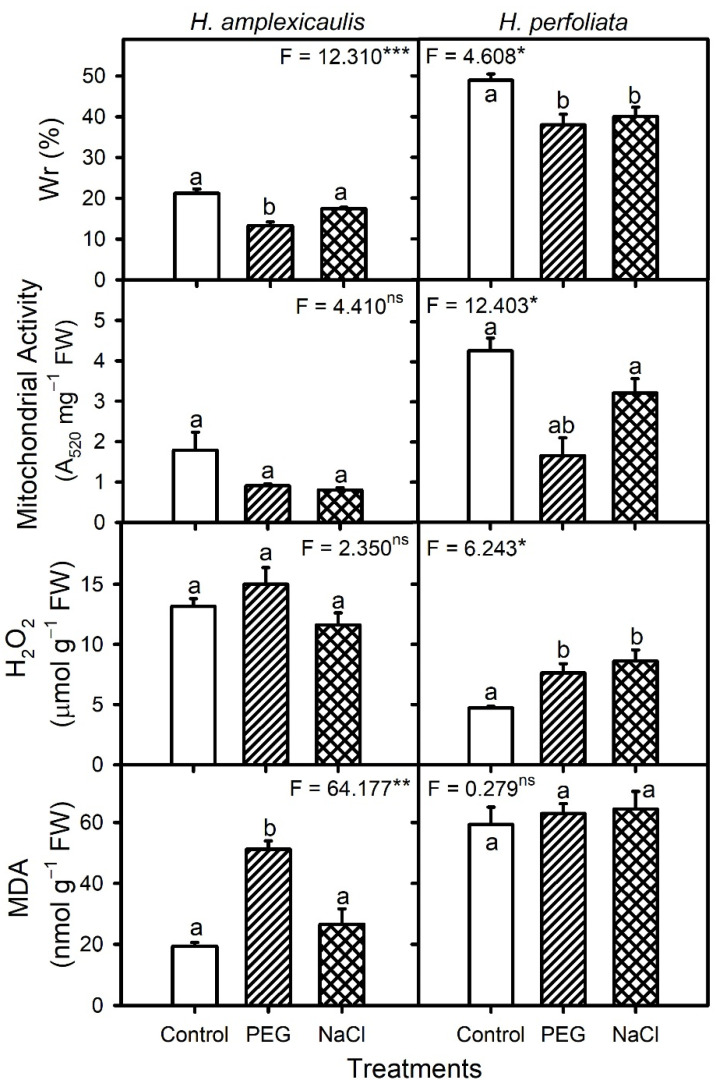
Relative water uptake (Wr), mitochondrial activity, hydrogen peroxide (H_2_O_2_) and malondialdehyde (MDA) contents of the germinating seeds of *H. amplexicaulis* and *H. perfoliata* under various treatments. Bars are mean ± standard error. Bars with different alphabets are significantly different from each other (LSD; *p* < 0.05). Superscripts over ANOVA F-values indicate level of significance, i.e., * = *p* < 0.05, ** = *p* < 0.01, *** = *p* < 0.001 and ns = *p* > 0.05.

Relative water uptake (Wr) of the seeds of two species decreased in the presence of NaCl and isotonic PEG treatments when compared to the unstressed control (Figure 9). Both NaCl and PEG treatments led to a comparable decline in Wr of the seeds of the test species. The Wr values of *H. perfoliata* seeds were significantly (*p* < 0.001; Table 10) higher than those of *H. amplexicaulis* (Figure 9). Mitochondrial activity of the *H. perfoliata*, but not that of the *H. amplexicaulis* seeds, decreased under stress conditions and *H. perfoliata* seeds displayed higher mitochondrial activity compared with *H. amplexicaulis* seeds (Figure 8). The hydrogen peroxide (H_2_O_2_, a common ROS) content of the germinating seeds of both species did not vary across different treatments; however, H. *amplexicaulis* seeds had a higher H_2_O_2_ content than that of *H. perfoliata* (Figure 8). In general, the H_2_O_2_ content negatively correlated to Wr (r = −0.842, *p* < 0.002) and mitochondrial activity (r = −0.737, *p* < 0.006). The malondialdehyde content of *H. amplexicaulis* seeds peaked in PEG compared with the control and NaCl treatments, whereas those of *H. perfoliata* seeds did not vary across the different treatments (Figure 9). 

**Figure 10:**
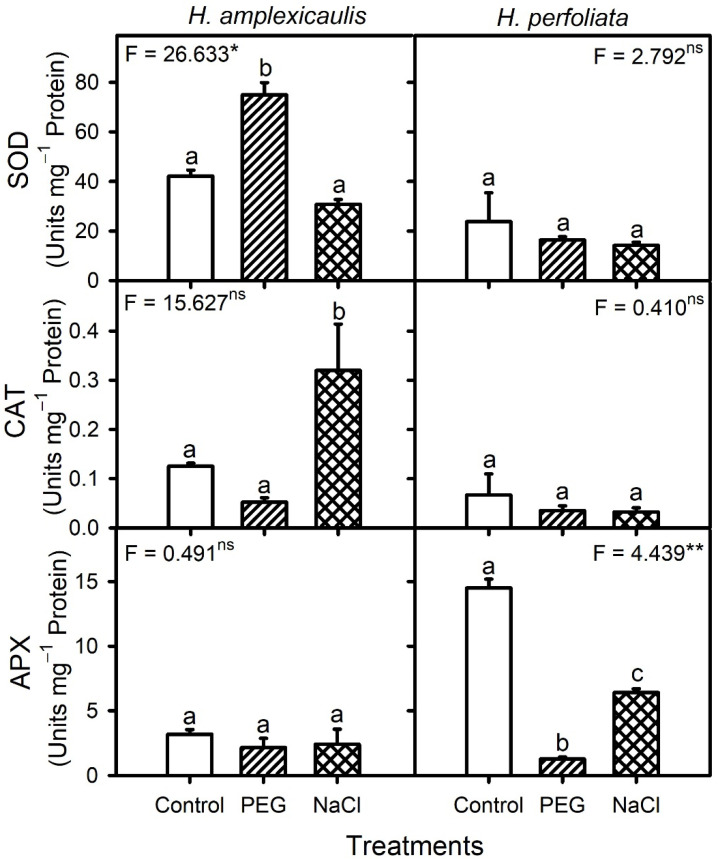
Activities of antioxidant enzymes in germinating seeds of *H. amplexicaulis* and *H. perfoliata* under various treatments. Bars are mean ± standard error. Bars with different alphabets are significantly different from each other (LSD; *p* < 0.05). Superscripts over ANOVA F-values indicate level of significance, i.e., * = *p* < 0.05, ** = *p* < 0.01, and ns = *p* > 0.05.

A two-way analysis of variance (ANOVA) indicated significant (*p* < 0.05) effects based on species (Sp), treatments (Tr) and their interaction with the antioxidant enzymes activities (Table 10). The activity of the SOD enzyme was higher in the presence of PEG compared with the control and NaCl in *H. amplexicaulis* seeds but was unaltered in *H. perfoliata* seeds across treatments (Figure 10). Overall, SOD activity had a positive correlation (r = 0.649, *p* < 0.012) with the H_2_O_2_ content of the seeds of the two species. The activity of the CAT enzyme in *H. amplexicaulis* seeds was highest in NaCl compared with the control and PEG, whereas that of *H. perfoliata* seeds did not vary among treatments (Figure 10). However, *H. amplexicaulis* seeds had significantly (*p* < 0.001) higher SOD and CAT levels in comparison with *H. perfoliata* seeds. In general, CAT activity had a negative correlation (r = −0.641, *p* < 0.007) with the MDA content of the seeds of test species. In contrast, *H. amplexicaulis* seeds had comparatively low and constitutive levels of APX activity when compared to *H. perfoliata* seeds, in which APX activity was lower under stress conditions than the control (Figure 10).

**Figure 11:**
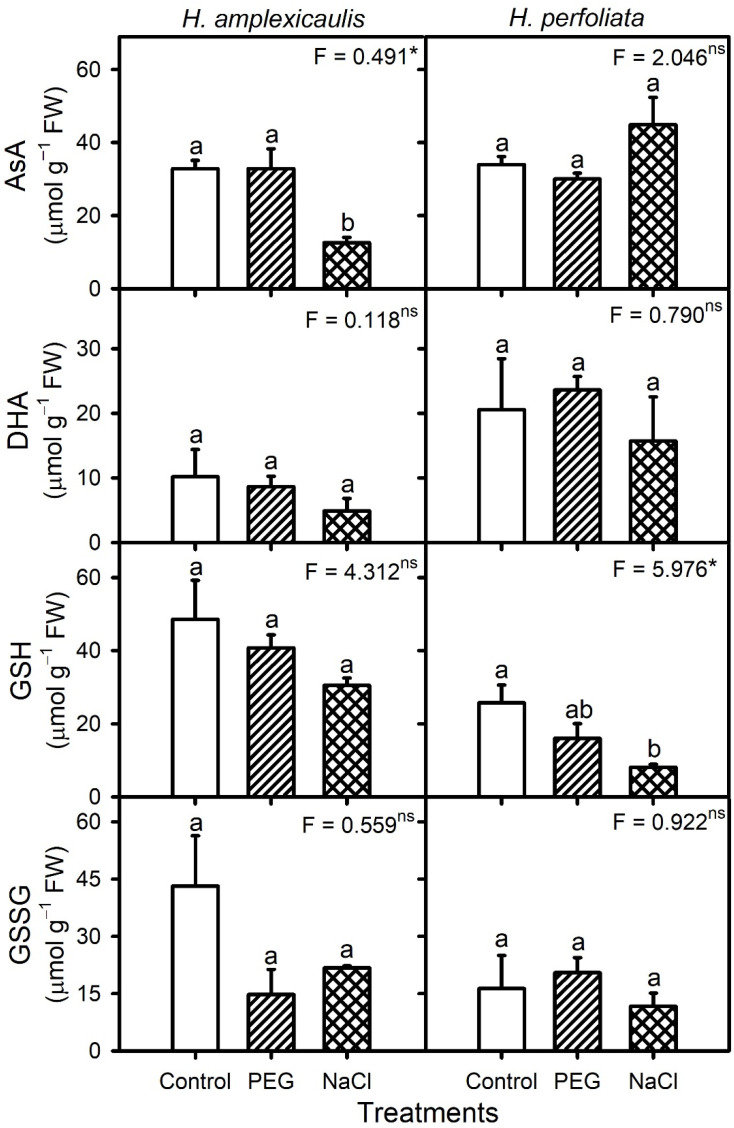
Levels of ascorbate (AsA and DHA) and glutathione (GSH and GSSG) in germinating seeds of *H. amplexicaulis* and *H. perfoliata* under various treatments. Bars are mean ± standard error. Bars with different alphabets are significantly different from each other (LSD; *p* < 0.05). Superscripts over ANOVA F-values indicate level of significance, i.e., * = *p* < 0.05, and ns = *p* > 0.05.

The ascorbate (AsA and DHA) contents of *H. perfoliata* seeds under isotonic NaCl and PEG were comparable to that in control whereas the AsA but not the DHA content of *H. amplexicaulis* seeds decreased in NaCl compared with the control and PEG (Figure 11). Seeds of *H. amplexicaulis* had comparatively higher and constitutive levels of GSH and GSSG than *H. perfoliata* seeds (Figure 10). NaCl treatment caused a reduction in the GSH content of *H. perfoliata* seeds compared with the control. In general, GSH activity had a negative correlation (r = −0.693, *p* < 0.004) with the MDA content of the seeds of the test species.

## 4. Discussion

Sensitivity to environmental fluctuations is an important physiological characteristic that allows seeds to germinate in specific environmental conditions [44,45]. Indeed, environmental regulation of germination is a multifaceted process that allows seeds to only germinate when environmental stresses do not surpass their limits of tolerance [46]. Among the two compared species, *H. perfoliata* reached high germination percentages at a wide range of temperatures, including the lowest temperature regime. This is a different result compared with other subtropical halophytes which prefer higher temperatures for germination [47,48], and is also an unexpected response for a plant living in a desert area with higher temperatures most of the year. This response could be a niche partitioning adaptation of the test species to confine its germination to the brief period of water availability rather than the temperature regime that regulates germination of most co-occurring species. The majority of rainfall in the natural habitat of *H. perfoliata* corresponds to the period of lower temperatures (from December to January), causing less soil water deficit and a less edaphic salt concentration, thus germinability under low temperatures will broaden the germination window of test species. Indeed, germination at the highest temperatures would be linked to osmotic stresses and therefore to a lower chance of success for seedlings. In this sense, Ref. [49] reported an increased germination percentage and rate at low temperature regimes in seeds of another halophyte, *Suaeda fruticosa,* under high maternal salinity. Comparatively, *H. amplexicaulis* reached high germination percentage at the intermediate temperature regimes (25/15 °C) that corresponds to the period of higher precipitation (April, September and October) in the growing area.

In many cases, salt tolerance under laboratory conditions may not correlate with plant responses under field conditions [50]. This fact could be explained in terms of the physiological responses to salinity, which are complex and vary with factors such as temperature, drought and soil texture. In the case of the two species of *Halopeplis* compared in this study, a higher tolerance was observed under laboratory conditions in *H. perfoliata*. This species reached higher germination percentages than those obtained in the tests carried out for the same species by other authors [51]. Nevertheless, salinity affected seed germination of both species, as reported in other species of the same family [52,53]. *Halopeplis perfoliata* reached a higher germination percentage in a shorter germination period, and it took less number of days until the first germination, which could be an adaptation to the environmental conditions of habitats with short and irregular rainfall periods that cause a decrease in soil salt concentration and reduce soil temperature, both suitable conditions for germination and seedling survival [51,54,55]. Moreover, a short germination period ensures an adequate quantity of seedlings under favourable conditions [56]. Most of the plants showing very fast germination grow in high-stress arid or saline habitats, and belong to the family Amaranthaceae, which is characterized by a generally higher salt tolerance in the germination phase [47]. The better germination percentage and speed in *H. perfoliata* seeds could also be ascribed to a maternal effect. A number of authors have observed that a maternal effect derived from the growth of plants in a saline environment can improve salt tolerance in the germination period, through a transmission of information to subsequent generations. This maternal effect has been observed in many halophytes [57,58], and their positive effect in improving a tolerance to high temperature regimes have also been probed in other species [49]. Conversely, some authors indicated better results in germination success for plants from non-saline environments when compared with plants growing in saline conditions [59]. Hence, effects of maternal environment on seed germination responses may vary among species.

Germination inhibition in halophytes, derived from an increase in salt concentration, can be the result of ionic toxicity and/or osmotic stress [60,61,62,63] depending on the species [64,65]. In this study, recovery data demonstrated that the inhibitory effect observed in PEG treatment is greater than that observed in NaCl in both species. These findings hint at the main role of osmotic stress in germination inhibition compared with specific ion effects. These results agree with those obtained in other species [48,66,67,68,69]. In this study, relative water uptake (Wr) data also indicated a similar decline under isotonic NaCl and PEG treatments compared with the control for both test species as reported for *Suaeda fruticosa* and *Limonium stocksii* [8]. This also hints at osmotic constraint for germination inhibition in test species under salt/osmotic solutions. However, Ref. [70] observed a greater inhibition of germination in seeds of several halophytes when treated with mannitol and/or PEG, compared with those treated with different concentrations of NaCl. Contrarily, in other species, such as *Suaeda heterophylla* [63] or *Atriplex halimus* [65] salinity caused ionic toxicity.

Reactive oxygen species (ROS) such as H_2_O_2_ are produced in germinating seeds during the imbibitional reactivation of mitochondrial oxygen metabolism and their production may enhance under environmental stresses such as salinity [8,12,71]. Such information about halophyte seeds is generally scant. In addition, only a few studies coupled biochemical analysis to explain the germination ecology responses of seeds. In this study, the H_2_O_2_ content of germinating seeds of two tested *Halopeplis* species in NaCl or PEG treatments were comparable to that in the control treatment. Likewise, the H_2_O_2_ content of brown seeds of *Arthrocnemum macrostachyum* and large seeds of *A. indicum* did not vary across salinity during germination [72]. The nearly static levels of H_2_O_2_ in halophyte seeds, including our test species, could be ascribed to unaltered mitochondrial activity, which also indicates that mitochondrial function was not compromised under NaCl/PEG treatment in the tested halophyte seeds. Unaltered or even decreased activities of H_2_O_2_ detoxification enzymes (CAT and APX) and non-enzymatic antioxidants (ascorbate and glutathione) in germinating seeds of both test species also hint at no excessive H_2_O_2_ production. Similarly, activities of antioxidant enzymes were either comparable or decreased under salinity compared with the control in germinating seeds of the halophyte *Gypsophila oblanceolate* [73]. Furthermore, the unaltered MDA (oxidative membrane damage marker) content of *H. perfoliata* in both NaCl and PEG compared with the control and that of *H. amplexicaulis* in NaCl compared with the control also indicate a lack of oxidative damage under stress conditions. Likewise, the MDA content of germinating seeds of halophytes *A. macrostachyum* [72] and *Salsola drummondii* [19] also did not increase under saline conditions. An increase in the MDA content of germinating *H. amplexicaulis* seeds in PEG solution, despite an unaltered H_2_O_2_ content, might be related to superoxide accumulation, which is indicated by the higher SOD activity. However, this increase in the MDA content was probably not too damaging, as most un-germinated seeds showed recovery of germination when transferred to the water. Hence, high recovery coupled with a lack of substantial oxidative damage indicates that germination inhibition under NaCl and isotonic PEG treatments in the two test halophytes probably resulted from a lack of sufficient imbibition owing to osmotic constraint rather than damages resulting from ionic toxicity.

Recovery tests showed a strong priming effect in *H. amplexicaulis*, while in *H. perfoliata* this effect was not noteworthy. Accordingly, some authors have reported better germination and an increased stress tolerance in seeds primed with NaCl or PEG, through a shortening of time for seed emergence, an increase in germination percentage and rate, an increase of chlorophyll content and root viability and the contribution in maintaining RWC under low soil moisture [74,75]. In this regard, plants living in saline environments have two different strategies (i.e., salt tolerance or salt avoidance), to survive in environments with high salt concentrations [4,76,77]. Salt avoidance mechanisms include phenological adaptations, such as short life cycles [78], and physiological adaptations, such as seed dormancy or germination timing. The high percentage of recovery germination after the alleviation of salinity corresponds to one of the salt-avoidance strategies of halophytes. This mechanism supplies a viable seed bank with new individuals able to germinate once the salinity decreases [79,80] and prevents seedling mortality in unfavourable periods [44]. Both strategies were found in the two studied species. *H. amplexicaulis* could be categorized as a salt-avoider species, compared with the salt-tolerant *H. perfoliata*, according to [81] who proved the use of one or both of these strategies in species belonging to the family Amaranthaceae. Moreover, the higher salt tolerance of *H. perfoliata* seems to be related to the climate conditions of its habitat (high temperature and low rainfall), that do not allow for a significant decrease in soil salinity concentration throughout the year.

Halophytes have different life-forms, from annuals to perennials, therefore one can expect different germination responses to salinity and drought depending on their life cycles. Accordingly, some authors demonstrated a greater salt tolerance in the germination phase for annual plants than in perennials and explained it in terms of their pioneer character, differences in the habitat colonized and the ability to complete the life cycles rapidly in an unpredictable environment [82]. In contrast, other authors identified annuals as the most sensitive to salinity [1], with stress escaping strategies [83,84]. Our results are in accordance with latter findings as they show a higher tolerance in the perennial *H. perfoliata* than in the annual *H. amplexicaulis*. In many cases, annual plants could be more demanding in terms of germination requirements, and their stress tolerance is lower because the survival of future generations depends exclusively on the survival of the seedlings after the germination of the seeds. According to the aforementioned results, it is expected that *H. perfoliata* will be less affected by global warming than *H. amplexicaulis*.

Research findings concerning seed size related to germination percentage and speed in stressful environments are not yet clear and conclusive enough [85]. However, generally a better adaptation to adverse conditions would be expected for larger seeds [86,87,88]. In this sense, Ref. [89] have evidenced a positive relationship between seed size and tolerance to water stress. However, some authors have evidenced opposing results [57,90], and in many cases, the absence of a relationship between seed size and germination response has been found [91,92]. Germination velocity can be affected in large seeds by the necessity of more water absorption and thus, a longer time for imbibition [85,93,94]. However, different authors have demonstrated fast germination in plants living in high stress environments as an adaptive mechanism that allows seeds to take advantage of short favourable periods and ensure the survival of seedlings [54,95]. In the case of the two *Halopeplis* species studied, there is no clear evidence of the relevance of this factor in germination response. According to our study and those from diverse authors, germination response in drought-affected habitats is more related to the environmental conditions of the growth area or genetic heterogeneity than other factors, e.g., seed size [92,96,97,98].

## 5. Conclusions

Our results provide new precise data proving the high adaptability of the germinative responses of *Halopeplis* species to local rainfall cycles. The data obtained indicates that the rainy period, linked to water availability, may act as a limiting factor which determines seed germination and conditioning in response to the temperature regime. Seed size does not appear to be a key factor in controlling germination response. The perennial species *H. perfoliata*, which comes from the hottest environment, could germinate in a wide range of temperature regimes. Whereas annual species *H. amplexicaulis* showed higher control of seed germination behaviour with a lower tolerance to osmotic stress. High and comparable recovery from NaCl and isotonic PEG treatments, alongside a lack of substantial oxidative damage, indicates that osmotic stress was responsible for the inhibitory effect observed in germination in both tested species under salinity. Halopriming, resulting from the limited hydration of ungerminated seeds in high salinity solutions, enhanced germination recovery of the annual species *H. amplexicaulis.* However, in light of its lower osmotic tolerance and a narrow temperature window, annual *H. amplexicaulis* could be more vulnerable to the climate change scenario. Since information about the biochemical basis of germination ecology responses under salt and thermal stresses of congeneric species with differing life-forms and from contrasting habitats is scarce, this study provides essential insights about the impacts of global changes on species abundance/distribution.

## Figures and Tables

**Table 1:** Seed traits of the two studied species and statistical significance (*t*-test).

	Length (mm)	Width (mm)	L/W	Weight (mg)
*H. perfoliata*	0.809 ± 0.062	0.585 ± 0.049	1.395 ± 0.170	0.127 ± 0.018
*H. amplexicaulis*	0.772 ± 0.044	0.610 ± 0.047	1.273 ± 0.116	0.134 ± 0.016
Sig. (*t*-test).	0.000	0.000	0.000	0.009

**Table 2:** Germination percentage (G%), first (FD) and last day (LD) of germination and germination period (GP) of the studied species in different temperature regimes and osmotic potentials (OP). Germinations ≤ 5% have not been considered to calculate other parameters.

		*H. amplexicaulis*				*H. perfoliata*			
T (°C)	OP	G %	FD	LD	GP	G %	FD	LD	GP
20/10	0	64.0 ± 19.6	3.5 ± 1.7	8.8 ± 0.5	5.3 ± 1.9	98.0 ± 19.6	1.0 ± 0.0	5.5 ± 1.0	4.5 ± 1.0
	−0.43	52.5 ± 10.6	4.0 ± 2.0	12.5 ± 2.4	8.5 ± 2.4	97.1 ± 10.6	1.0 ± 0.0	3.3 ± 0.5	2.3 ± 0.5
	−0.85	45.0 ± 13.2	7.0 ± 1.8	15.0 ± 2.0	8.0 ± 2.2	99.0 ± 13.2	1.0 ± 0.0	5.3 ± 0.5	4.3 ± 0.5
	−1.28	11.0 ± 6.8	10.0 ± 2.7	14.8 ± 1.0	4.8 ± 3.3	95.0 ± 6.8	3.0 ± 0.0	6.5 ± 0.6	3.5 ± 0.6
	−1.70	3.0 ± 3.8	-	-	-	57.0 ± 3.8	5.5 ± 1.0	8.5 ± 1.0	3.0 ± 1.2
	−2.13	0.0 ± 0.0	-	-	-	0.0 ± 0.0	-	-	-
	−2.55	0.0 ± 0.0	-	-	-	0.0 ± 0.0	-	-	-
25/15	0	88.0 ± 3.3	2.3 ± 1.0	9.5 ± 5.9	7.3 ± 6.0	92.0 ± 3.3	1.0 ± 0.0	5.0 ± 1.2	4.0 ± 1.2
	−0.43	81.0 ± 11.9	3.8 ± 0.5	15.0 ± 6.9	11.3 ± 6.7	98.0 ± 11.9	1.0 ± 0.0	5.8 ± 0.5	4.8 ± 0.5
	−0.86	77.0 ± 5.0	4.5 ± 1.0	10.5 ± 3.1	6.0 ± 3.2	97.0 ± 5.0	3.5 ± 1.0	7.8 ± 3.5	4.3 ± 2.5
	−1.30	42.0 ± 12.4	3.8 ± 3.5	15.5 ± 1.7	11.8 ± 3.6	62.0 ± 12.4	5.5 ± 3.3	9.5 ± 4.0	4.0 ± 0.8
	−1.73	6.0 ± 5.2	9.7 ± 3.8	15.3 ± 6.7	4.3 ± 6.7	21.0 ± 5.2	13.0 ± 5.8	16.0 ± 5.4	3.0 ± 4.8
	−2.16	0.0 ± 0.0	-	-	-	1.0 ± 0.0			
	−2.59	0.0 ± 0.0	-	-	-	0.0 ± 0.0	-	-	-
30/20	0	82.5 ± 9.6	2.3 ± 0.5	19.0 ± 2.0	16.8 ± 1.9	100.0 ± 9.6	1.0 ± 0.0	5.0 ± 0.0	4.0 ± 0.0
	−0.44	46.3 ± 15.5	4.0 ± 1.4	12.5 ± 4.7	8.5 ± 4.1	97.0 ± 15.5	1.0 ± 0.0	6.0 ± 0.0	5.0 ± 0.0
	−0.88	0.0 ± 0.0	-	-	-	87.0 ± 0.0	2.3 ± 1.3	7.5 ± 1.7	5.3 ± 1.3
	−1.32	0.0 ± 0.0	-	-	-	78.0 ± 0.0	5.8 ± 0.5	9.3 ± 1.0	3.5 ± 1.3
	−1.76	1.3 ± 2.5	-	-	-	4.0 ± 2.5	-	-	-
	−2.20	0.0 ± 0.0	-	-	-	1.0 ± 0.0	-	-	-
	−2.64	0.0 ± 0.0	-	-	-	0.0 ± 0.0	-	-	-
35/25	0	32.5 ± 17.1	4.8 ± 3.2	18.0 ± 1.8	13.3 ± 1.7	97.0 ± 17.1	1.0 ± 0.0	5.5 ± 1.0	4.5 ± 1.0
	−0.45	6.3 ± 6.3	17.7 ± 4.0	19.7 ± 0.6	1.5 ± 3.0	31.0 ± 6.3	6.0 ± 0.0	8.0 ± 0.0	2.0 ± 0.0
	−0.89	5.0 ± 0.0	-	-	-	5.0 ± 0.0	-	-	-
	−1.34	1.3 ± 2.5	-	-	-	0.0 ± 2.5	-	-	-
	−1.79	0.0 ± 0.0	-	-	-	0.0 ± 0.0	-	-	-
	−2.24	1.1 ± 2.2	-	-	-	0.0 ± 2.2	-	-	-
	−2.68	1.3 ± 2.5	-	-	-	0.0 ± 2.5	-	-	-

**Table 3:** Recovery of germination tests. Germination percentage (G %), first (FD) and last day (LD) of germination and germination period (GP) of the studied species in different temperature regimes and different osmotic potential (OP) obtained through increasing salt concentrations. Germinations values of ≤5% were not considered to calculate other parameters.

		*H. amplexicaulis*				*H. perfoliata*			
T (°C)	OP	G %	FG	LD	GP	G %	FD	LD	GP
20/10	−0.43	38.4 ± 13.8	1.8 ± 1.0	9.8 ± 5.5	8.0 ± 6.4	0.0 ± 0.0	-	-	-
	−0.85	87.1 ± 11.2	1.0 ± 0.0	6.3 ± 1.5	5.3 ± 1.5	0.0 ± 0.0	-	-	-
	−1.28	88.2 ± 9.1	1.0 ± 0.0	4.0 ± 0.0	3.0 ± 0.0	25.0 ± 50.0	1.0	1.0	0.0
	−1.70	93.8 ± 5.2	1.0 ± 0.0	4.8 ± 1.5	3.8 ± 1.5	89.1 ± 8.0	1.0 ± 0.0	1.0 ± 0.0	0.0
	−2.13	95.8 ± 3.4	1.0 ± 0.0	3.8 ± 1.3	2.8 ± 1.3	98.0 ± 4.0	1.0 ± 0.0	2.3 ± 1.9	1.3 ± 1.9
	−2.55	99.0 ± 2.0	2.5 ± 0.6	4.3 ± 1.9	1.8 ± 2.2	97.0 ± 2.0	1.0 ± 0.0	5.3 ± 2.9	4.3 ± 2.9
25/15	−0.43	81.3 ± 23.9	1.0 ± 0.0	2.3 ± 0.5	1.3 ± 0.5	0.0 ± 0.0	-	-	-
	−0.85	96.4 ± 7.1	1.0 ± 0.0	2.0 ± 0.0	1.0 ± 0.0	33.3 ± 57.7	1.0	1.0	0.0
	−1.28	100.0 ± 0.0	1.0 ± 0.0	2.5 ± 0.6	1.5 ± 0.6	94.4 ± 11.1	1.0 ± 0.0	2.5 ± 3.0	1.5 ± 3.0
	−1.70	98.9 ± 2.2	1.0 ± 0.0	2.3 ± 0.5	1.3 ± 0.5	91.4 ± 7.8	1.0 ± 0.0	2.0 ± 2.0	1.0 ± 2.0
	−2.13	98.0 ± 4.0	1.0 ± 0.0	2.3 ± 0.5	1.3 ± 0.5	91.0 ± 5.0	1.0 ± 0.0	1.0 ± 0.0	0.0
	−2.55	100.0 ± 0.0	1.3 ± 0.5	5.0 ± 3.6	3.8 ± 3.9	90.0 ± 10.6	1.0 ± 0.0	4.0 ± 3.5	3.0 ± 3.5
30/20	−0.43	100.0 ± 0.0	1.0 ± 0.0	2.0 ± 0.0	1.0 ± 0.0	0.0 ± 0.0	-	-	-
	−0.85	100.0 ± 0.0	1.3 ± 0.5	6.3 ± 5.9	5.0 ± 5.4	10.7 ± 21.4	1.0	9.0	8.0
	−1.28	97.5 ± 5.0	1.0 ± 0.0	2.8 ± 1.0	1.8 ± 1.0	34.4 ± 23.7	1.0 ± 0.0	1.0 ± 0.0	0.0
	−1.70	100.0 ± 0.0	1.0 ± 0.0	4.3 ± 2.1	3.3 ± 2.1	75.9 ± 12.7	1.0 ± 0.0	7.0 ± 4.0	6.0 ± 4.0
	−2.13	96.3 ± 7.5	1.0 ± 0.0	5.5 ± 1.7	4.5 ± 1.7	62.8 ± 24.1	1.0 ± 0.0	9.3 ± 0.5	8.3 ± 0.5
	−2.55	95.0 ± 7.1	1.0 ± 0.0	1.8 ± 0.5	0.8 ± 0.5	46.0 ± 14.8	1.0 ± 0.0	5.5 ± 5.2	4.5 ± 5.2
35/25	−0.43	98.4 ± 3.1	1.0 ± 0.0	7.0 ± 6.9	6.0 ± 6.9	28.4 ± 21.0	1.0 ± 0.0	5.7 ± 0.6	4.7 ± 0.6
	−0.85	98.7 ± 2.6	1.0 ± 0.0	3.8 ± 2.2	2.8 ± 2.2	3.3 ± 4.2	-	-	-
	−1.28	93.6 ± 6.7	1.0 ± 0.0	11.8 ± 6.7	10.8 ± 6.7	5.0 ± 3.8	-	-	-
	−1.70	86.3 ± 10.3	1.0 ± 0.0	14.3 ± 3.5	13.3 ± 3.5	0.0 ± 0.0	-	-	-
	−2.13	81.3 ± 16.5	1.0 ± 0.0	10.3 ± 6.7	9.3 ± 6.7	1.0 ± 2.0	-	-	-
	−2.55	70.1 ± 6.6	1.0 ± 0.0	2.5 ± 0.6	1.5 ± 0.6	1.0 ± 2.0	-	-	-

**Table 4:** Recovery germination rate, (mean ± SE) of *Halopeplis amplexicaulis* seeds under increasing salt concentration at the different temperature regimes tested (*p* < 0.05). Germinations values of ≤5% were not considered to calculate this parameter. Values with different letters are significantly different from each other within rows (*p* < 0.05).

	Osmotic Potential (MPa)						
T (°C)	0	−0.43 to −0.45	−0.85 to −0.89	−1.28 to −1.34	−1.70 to −1.79	−2.13 to −2.24	−2.55 to −2.68
20/10	50.6 ± 15.7 b	34.5 ± 11.5 b	76.4 ± 8.4 a	82.2 ± 9.4 a	91.0 ± 6.1 a	92.9 ± 3.3 a	96.1 ± 2.2 a
25/15	75.0 ± 6.1 b	75.9 ± 18.6 b	95.8 ± 6.8 a	100.0 ± 0.0 a	98.7 ± 2.0 a	97.9 ± 3.9 a	99.9 ± 0.2 a
30/20	52.0 ± 3.9 b	100.0 ± 0.0 a	100.0 ± 0.0 a	94.0 ± 8.7 a	99.5 ± 0.7 a	93.6 ± 9.6 a	91.8 ± 7.3 a
35/25	14.6 ± 5.4 c	97.1 ± 5.1 a	96.6 ± 3.8 a	90.6 ± 8.1 a	80.8 ± 13.7 ab	70.8 ± 11.1 b	64.1 ± 7.7 b

**Table 5:** Recovery germination rate, (mean ± SE) of *Halopeplis perfoliata* seeds under increasing salt concentrations at the different temperature regimes tested (*p* < 0.05). Values with different letters are significantly different from each other within rows (*p* < 0.05).

	Osmotic Potential (MPa)						
T (°C)	0	−0.43 to −0.45	−0.85 to −0.89	−1.28 to −1.34	−1.70 to −1.79	−2.13 to −2.24	−2.55 to −2.68
20/10	90.5 ± 4.1 a	0.0 c	0.0 c	2.0 ± 4.0 c	38.0 ± 4.0 b	98.0 ± 4.0 a	95.0 ± 5.0 a
25/15	86.0 ± 8.2 a	0.0 c	1.3 ± 2.3 c	33.5 ± 17.1 b	70.5 ± 20.6 a	90.0 ± 4.0 a	89.0 ± 10.4 a
30/20	97.0 ± 2.0 a	0.0 c	2.0 ± 4.0 c	7.0 ± 5.0 c	70.5 ± 16.8 ab	57.5 ± 21.5 b	44.0 ± 16.7 b
35/25	92.0 ± 4.6 a	17.5 ± 14.7 b	-	-	-	-	-

**Table 6:** Recovery germination rate (mean ± SE) of *Halopeplis amplexicaulis* seeds under increasing PEG concentrations at the different temperature regimes tested. Germinations values of ≤5% were not considered to calculate this parameter. Values with different letters are significantly different from each other within rows (*p* < 0.05).

	Osmotic Potential (MPa)						
T (°C)	0	−0.43 to −0.45	−0.85 to −0.89	−1.28 to −1.34	−1.70 to −1.79	−2.13 to −2.24	−2.55 to −2.68
20/10	50.6 ± 15.7 a	57.5 ± 10.6 a	58.0 ± 40.2 a	81.0 ± 6.6 a	73.5 ± 20.8 a	68.9 ± 14.8 a	79.5 ± 6.5 a
25/15	75.0 ± 6.1 a	91.2 ± 9.7 a	98.7 ± 1.6 a	98.9 ± 1.4 a	78.8 ± 25.1 a	78.0 ± 12.7 a	80.5 ± 8.0 a
30/20	52.0 ± 3.9 b	97.0 ± 5.4 a	97.4 ± 4.9 a	98.3 ± 2.8 a	98.3 ± 2.2 a	96.9 ± 3.7 a	93.0 ± 4.6 a
35/25	14.6 ± 5.4 b	75.5 ± 17.1 a	48.3 ± 25.0 ab	42.9 ± 23.3 ab	46.8 ± 9.9 ab	29.4 ± 26.1 b	17.9 ± 6.9 b

**Table 7:** Recovery germination rate (mean ± SE) of *Halopeplis perfoliata* seeds under increasing PEG concentrations at the different temperature regimes tested. Values with different letters are significantly different from each other within rows (*p* < 0.05).

	Osmotic Potential (MPa)						
T (°C)	0	−0.43 to −0.45	−0.85 to −0.89	−1.28 to −1.34	−1.70 to −1.79	−2.13 to −2.24	−2.55 to −2.68
20/10	90.5 ± 4.1 a	13.0 ± 1.2 d	6.0 ± 3.3 d	60.0 ± 12.3 b	71.0 ± 2.6 b	45.5 ± 1.0 c	38.5 ± 5.3 c
25/15	86.0 ± 8.2 a	2.7 ± 4.6 b	1.0 ± 2.0 b	5.0 ± 4.2 b	90.0 ± 5.9 a	91.5 ± 6.4 a	85.0 ± 7.4 a
30/20	97.0 ± 2.0 a	0.0 c	0.0 c	6.5 ± 1.9 c	56.0 ± 20.9 b	92.0 ± 7.3 a	88.5 ± 3.4 a
35/25	92.0 ± 4.6 a	-	-	-	84.5 ± 16.8 a	82.5 ± 13.3 a	-

**Table 8:** Tolerance Index (TI) at the different osmotic potential (OP) of the salt and PEG solutions. Ha: *H. amplexicaulis*; Hp: *H. perfoliata*.

T	OP (MPa)	Ha NaCl	Hp NaCl	Ha PEG	Hp PEG
20/10 °C	−0.43	0.82	0.99	0.00	0.79
	−0.85	0.70	1.01	0.00	0.70
	−1.28	0.17	0.97	0.00	0.29
	−1.70	0.05	0.58	0.00	0.00
	−2.13	0.00	0.00	0.00	0.00
	−2.55	0.00	0.00	0.00	0.00
25/15 °C	−0.43	0.92	1.07	0.99	1.04
	−0.86	0.88	1.05	0.57	1.03
	−1.30	0.48	0.67	0.00	0.00
	−1.73	0.07	0.23	0.00	0.00
	−2.16	0.00	0.01	0.00	0.00
	−2.59	0.00	0.00	0.00	0.00
30/20 °C	−0.44	0.56	0.97	0.42	0.98
	−0.88	0.00	0.87	0.02	0.92
	−1.32	0.00	0.78	0.00	0.00
	−1.76	0.02	0.04	0.00	0.35
	−2.20	0.00	0.01	0.00	0.00
	−2.64	0.00	0.00	0.00	0.00
35/25 °C	−0.45	0.19	0.32	0.00	0.99
	−0.89	0.15	0.05	0.00	0.77
	−1.34	0.04	0.00	0.00	0.00
	−1.79	0.00	0.00	0.00	0.00
	−2.24	0.03	0.00	0.00	0.00
	−2.68	0.04	0.00	0.00	0.00

**Table 9:** F-values from a three-way ANOVA testing the effect of species, salt treatments, temperature and their interactions on germination percentage (on salt (S) and the recovery (R)) and germination rate (GR). Differences between means were considered to be significant at *p* < 0.05.

	G	GR (S)	R	IT(R)
Variable	*F*	*F*	*F*	*F*
Species	213.8	437.5	183.1	694.6
Temperature	174.9	236.3	41.3	97.5
[NaCl]	475.8	601.1	23.3	79.3
Species × Temperature	38.7	65.3	17.5	28.1
Species × [NaCl]	34.7	80.8	47.8	210.9
Temperature × [NaCl]	24.7	34.4	11.5	31.0
Species × Temperature × [NaCl]	13.5	20.9	4.6	13.9

*p* = 0.000 in all cases.

**Table 10:** Two-way ANOVA indicating effects of species (Sp), treatments (Tr) and their interaction on various biochemical parameters of the germinating seeds of two *Halopeplis* species. Numbers are F-values and asterisks (*) are *p* values (Where, ns = *p* > 0.05, * = *p* < 0.05, and *** = *p* < 0.001).

Parameters	Sp	Tr	Sp × Tr
Wr	307.12 ***	12.15 ***	0.19 ^ns^
Mitochondrial activity	51.78 ***	14.99 ***	4.69 ^ns^
H_2_O_2_	42.91 ***	1.30 ^ns^	2.31 ^ns^
MDA	76.51 ***	9.26 ***	7.04 ***
SOD	30.20 ***	7.38 *	9.53 ***
CAT	16.04 ***	6.54 *	8.55 ***
APX	14.74 ***	4.90 *	3.25 ^ns^
AsA	3.38 ^ns^	0.42 ^ns^	1.57 ^ns^
DHA	5.07 *	0.47 ^ns^	0.73 ^ns^
GSH	44.47 ***	8.80 ***	1.33 ^ns^
GSSG	2.47 ^ns^	2.26 ^ns^	2.22 ^ns^

## Data Availability

Not applicable.
